# Endoplasmic reticulum stress mediating downregulated StAR and 3-beta-HSD and low plasma testosterone caused by hypoxia is attenuated by CPU86017-RS and nifedipine

**DOI:** 10.1186/1423-0127-19-4

**Published:** 2012-01-08

**Authors:** Gui-Lai Liu, Feng Yu, De-Zai Dai, Guo-Lin Zhang, Can Zhang, Yin Dai

**Affiliations:** 1 Faculty of Pharmacy, China Pharmaceutical University, Nanjing, 210009, China

**Keywords:** ER stress, testosterone, hypoxia, StAR, 3-beta-HSD, CHOP, PERK, Bip, testes, CPU86017

## Abstract

**Background:**

Hypoxia exposure initiates low serum testosterone levels that could be attributed to downregulated androgen biosynthesizing genes such as StAR (steroidogenic acute regulatory protein) and 3-beta-HSD (3-beta-hydroxysteroid dehydrogenase) in the testis. It was hypothesized that these abnormalities in the testis by hypoxia are associated with oxidative stress and an increase in chaperones of endoplasmic reticulum stress (ER stress) and ER stress could be modulated by a reduction in calcium influx. Therefore, we verify that if an application of CPU86017-RS (simplified as RS, a derivative to berberine) could alleviate the ER stress and depressed gene expressions of StAR and 3-beta-HSD, and low plasma testosterone in hypoxic rats, these were compared with those of nifedipine.

**Methods:**

Adult male Sprague-Dawley rats were randomly divided into control, hypoxia for 28 days, and hypoxia treated (mg/kg, p.o.) during the last 14 days with nifedipine (Nif, 10) and three doses of RS (20, 40, 80), and normal rats treated with RS isomer (80). Serum testosterone (T) and luteinizing hormone (LH) were measured. The testicular expressions of biomarkers including StAR, 3-beta-HSD, immunoglobulin heavy chain binding protein (Bip), double-strand RNA-activated protein kinase-like ER kinase (PERK) and pro-apoptotic transcription factor C/EBP homologous protein (CHOP) were measured.

**Results:**

In hypoxic rats, serum testosterone levels decreased and mRNA and protein expressions of the testosterone biosynthesis related genes, StAR and 3-beta-HSD were downregulated. These changes were linked to an increase in oxidants and upregulated ER stress chaperones: Bip, PERK, CHOP and distorted histological structure of the seminiferous tubules in the testis. These abnormalities were attenuated significantly by CPU86017-RS and nifedipine.

**Conclusion:**

Downregulated StAR and 3-beta-HSD significantly contribute to low testosterone in hypoxic rats and is associated with ER stress which mediates testis damage caused by oxygen deprivation. CPU86017-RS is potential in ameliorating hypoxia-induced testicular injuries, possibly by its calcium antagonist effects on the testis.

## Background

Male hypogonadism is defined as low serum testosterone under 300 ng/dL that has been considered as one of the major concerns in the modern society [1]. Regarding the possible mechanisms underlying, oxidative stress in the testis serves as the main causal factor actively involved in the pathogenesis of male hypogonadism [2]. Among various etiologies, hypoxia causes oxygen deprivation in the testis contributing to reduced production of androgen, in which a combination with pro-inflammatory factors including ET-1 (endothelin -1), leptin and ROS in initiating testicular abnormalities is likely involved [3]. Oxygen deprivation induces a series of mitochondria dysfunction facilitating an increase of oxidants and a decrease of antioxidants, resulting in an alteration of the redox system, then, oxidative stress occurs [4,5]. In the oxygen deprived condition, an increase in transcription promoting factors exacerbates the production of inflammatory and pro-inflammatory cytokines likely caused by hypoxia. Therefore, a low level of inflammation exists in the testis affecting adversely expressions of the androgen production genes such as StAR and 3-beta-HSD. The low inflammatory situation is always characterized by increasing chaperones of endoplasmic reticulum stress (ER stress). Endoplasmic reticulum (ER), a membrane-enclosed reticular network, is the site for the maturation of newly synthesized proteins requiring appropriate folding process through various spatial configurations [6]. Hypoxia causes an excess of oxidants including both ROS (reactive oxygen species) and RNS (reactive nitrogen species) interfering with the process of unfolded protein response (UPR) [7]. At the beginning, UPR is favorable for cell-protecting activity possibly leading to a relief to ER stress. Along with the further development of ER stress, the signals such as IRE1 (inositol-requiring enzyme-1), PERK (double-strand RNA-activated protein kinase-like ER kinase), ATF6 (activating transcription factor-6) and CHOP (C/EBP homologous protein) are exaggerated, then, the dialogue between the endoplasmic reticulum and the nucleus is altered, triggering the signaling cascades to induce adverse events in cells, finally resulting in apoptosis, cell death and disease [8].

Stress, stimulated by oxygen deprivation, adversely affects the testis by reducing sperm genesis, histological changes associated with low testosterone production [9,10]. In our previous reports hypoxic pulmonary hypertension (HPH) where oxidative stress develops is subsequent to an activation of the ET-ROS pathway [11,12]. Downregulation of FKBP12.6 and SERCA2a at the endoplasmic reticulum (ER) in cardiac myocytes is associated with an upregulated endothelin (ET) system facilitating ROS genesis [13,14]. Exaggerated production of ROS causes telomere shortened, spermatogenesis decreased and testosterone biosynthesis reduced, these abnormalities are likely to happen in aging [15]. Disturbance of ER function regarding calcium homeostasis induces abnormal protein folding process through ER stress and the UPR [16]. Eventually, ER stress facilitates the appearance of apoptosis through activating calcium homeostasis and exaggerated ROS production, accounting for cell dysfunction, insults and death [17]. However, it is unclear if hypoxia induced downregulated StAR and 3-beta-HSD and low testosterone in plasma are due to an involvement of ER stress in the hypoxia testis and these abnormalities could be blunted by interventions with calcium antagonism.

CPU86017, a derivative of berberine, has been reported to have calcium antagonism, α-adrenoceptor blocking effects and antioxidative activities [18]. CPU86017 is a racemate, containing two chiral centers: 7N and 13aC, and the 4 isomers are active in suppressing L-type channels similar to those of the racemate. Among them, the activity of RS isomer is most favorable in treating hypoxic pulmonary hypertension [19].

We hypothesized that ER stress might be actively implicated in the hypoxic testicular injuries likely mediated by an increase in calcium influx which is augmented on hypoxia [20]. Thus, a blockade on the calcium influx by nifedipine and CPU86017-RS might be beneficial in normalizing StAR, 3-beta-HSD, low testosterone, and the ER stress due to oxygen deprivation. This study was aimed to verify whether ER stress is responsible for low expression of StAR and 3-beta-HSD, and low testosterone involved in hypoxia-damaged testes and could be blunted by CPU86017-RS, compared to those of nifedipine.

## Materials and methods

### Materials

CPU86017-RS (RS), was synthesized by the Center of New Drug Discovery, China Pharmaceutical University and nifedipine (Nif) was purchased from Kangpu Drug Manufacturer, Changzhou, China. The MMLV RT (Moloney Murine Leukemia Virus Reverse Transcriptase; Promega, Madison, WI) and Taq DNA polymerase (Tiangen Biotech, Beijing, China) were purchased from Tianwei Company, Nanjing, China. Monoclonal mouse anti-StAR-IgG, anti-CHOP-IgG, polyclonal rabbit anti-PERK-IgG and anti-Bip-IgG were purchased from Santa Cruz Biotechnology (Santa Cruz, CA); polyclonal rabbit anti-3-beta-HSD-IgG from Novus Biotechnology; HRP-conjugated polyclonal goat anti-mouse IgG and polyclonal goat anti-rabbit IgG from Boster Biological Technology, Wuhan, China

### Animals

Adult male Sprague-Dawley rats, weighing 200-220 g, were obtained from The Zhejiang Experimental Animal Center in Hangzhou, Zhejiang Province, with a license No. SCXK20080033. The treatment of rats was strictly conformed to the Guideline of Handling Experimental Animals set up by the Science-Technology Bureau of Jiangsu Province, China.

### Hypoxia and Treatment

Rats were randomly divided into seven groups (n = 10): control, hypoxia for 28 days, and hypoxia treated (mg/kg, p.o.) during the last 14 days with Nif (10) and 3 doses of RS isomer (20, 40, 80), and normal rats treated with RS isomer (80). Hypoxia exposure was conducted according to the previous studies with some modifications [11,19]. Briefly, rats were housed in a normobaric chamber 8 h per day, and inside O_2 _concentration at 10 ± 0.5% controlled by driving in N_2 _with an instant monitoring system. The hypoxia condition is equivalent to 6 000 m highland. Rats were under hypoxia sustained for 4 weeks. Sufficient amount of soda lime and anhydrous calcium chloride was placed inside to absorb unnecessary CO_2 _and moisture. Rats in control and untreated groups were received an equal volume of 0.5% carboxy-methyl-cellulose Na (CMC-Na).

### Histological evaluation

The testicular tissue was fixed with 10% neutral formalin, embedded in paraffin, and sliced into 5-mm-thick pieces that were stained with routine hematoxylin-eosin (H-E) staining. The examination of all slices was conducted under light microscopy by a pathologist blinded to the experimental profile, and pictures were taken at ×200 amplification [21].

### Biochemical assays

Serum testosterone (T) and luteinizing hormone (LH) were conducted by applying chemiluminescence assay as in previous reports [21]. The contents of malondialdehyde (MDA) and activities of glutathione peroxidase (GSH-px) and lactate dehydrogenase (LDH) were assayed following instructions of the kits provided by the Nanjing Jiancheng Bio-engineering Institute (China).

### Reverse transcription PCR

The mRNA abundance of StAR, 3-beta-HSD, Bip, PERK and CHOP was measured by RT-PCR according to the previous reports [19]. Briefly, total RNA extraction from testicular tissue by Trizol reagent was reversely transcribed into cDNA using MMLV RT according to the manufacturer's introduction. RT-PCR was performed in a volume of 25 uL with a 1-μg aliquot of cDNA, and the products were stained with ethidium bromide and detected under an ultraviolet lamp (GDS8000; Sygene, Cambridge, UK). The densitometry of each band was analyzed using professional image analysis software, and the ratio of the target gene against the GAPDH internal standard was calculated. The nucleotide sequences of primers and PCR amplification conditions are listed in Table 1.

**Table 1:** Sequence of primers and conditions used for RT-PCR amplification

Primers	Sequence of primers	PCR amplification conditions
StAR	Sense:5'-CTCAACAACCAAGGAAGGCTGG-3'	94°C, 60 s; 56°C, 40 s;
	Atisense:5'-GCAGGTGGGGCCGTGTTCAGC-3'	72°C, 40 s; 30 cycles
3-beta-HSD	Sense:5'-ACTGGCAAATTCTCCATAGCC-3'	94°C, 30 s; 60°C, 45 s;
	Atisense:5'-GCTGAACAGTCGACCCTCCTT-3'	72°C, 60 s; 30 cycles
CHOP	Sense:5'-TCTGCCTTTCGCCTTTGAG-3'	94°C, 40 s; 55°C, 60 s;
	Atisense:5'-GCTTTGGGAGGTGCTTGTG-3'	72°C, 40 s; 29 cycles
Bip	Sense:5'-CATCAATGAGCCAACAGC-3'	94°C, 45 s; 60°C, 60 s;
	Atisense: 5'-AGGTAGAGCGGAACAGG-3'	72°C, 45 s; 26 cycles
PERK	Sense: 5'-GCCGATGGGATAGTGATG-3'	94°C, 45 s; 62°C, 60 s;
	Atisense: 5'-GCAGCCTCTACAATGTCTTCT-3'	72°C, 45 s; 28 cycles
GAPDH	Sense:5'-GCTGGGGCTCACCTGAAGG-3'	94°C, 45 s; 56°C, 60 s;
	Atisense:5'-GGATGACCTTGCCCACAGCC-3'	72°C, 60 s; 30 cycles

### Western blot analysis

To conduct quantitative analysis of protein levels of StAR, 3-beta-HSD, Bip, PERK and CHOP, a portion of testicular tissue (100 mg) was homogenized in four volumes of extraction buffer and then centrifuged at 10,000×g at 4°C (for 10 min. After the protein concentrations were determined, the supernatant was stored at -20°C before use. Aliquots of samples were heated to 98°C in a loading buffer and fractionated on 10% sodium dodecyl sulfate polyacrylamide gel electrophoresis (SDS-PAGE). Following the transfer to a nitrocellulose membrane and blocking with nonfat milk (5%, w/v), the blot was incubated at 4°C overnight with specific primary antibody. Three washes later, the blot incubated with horseradish peroxidase (HRP)-conjugated goat secondary antibody IgG (Afﬁnity Bioreagents; 1:1000) for 1 h at room temperature was detected with a DAB kit. The bands was visualized by an imaging acquisition (Labworks, UK) and quantified by densitometry. The relative abundance was obtained by normalizing the density of the tested proteins against that of β-actin [22].

### Statistical analysis

All data were analyzed with SPSS 11.5 (USA) and presented as the means ± SD. Statistical evaluation was conducted by one-way ANOVA. Then Bonferroni multiple comparison tests were applied to check the signiﬁcance of differences; in checking the variances of two independent samples an Independent-Sample t-test was used. A probability value of *P *< 0.05 was considered to be statistically significant.

## Results

### Testosterone and LH

**Figure 1:**
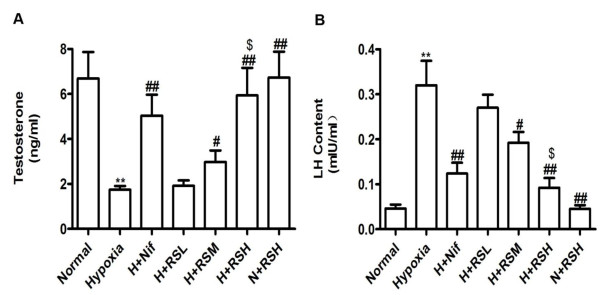
**Serum testosterone and LH levels**. A reduction in testosterone and an elevated LH in serum were found in rats explored to intermittent hypoxia for 4 weeks, and these changes were relieved by medication (mg/kg, po) with CPU86017-RS (20, 40, 80, termed as RSL, RSM, RSH) and Nif (10) in the last 2 weeks. A, Testosterone, and B, LH. Mean ± SD, n = 10. *P < 0.05, **P < 0.01, *vs*. Normal; ^#^*P *< 0.05, ^# #^*P *< 0.01, *vs*. Hypoxia, ^$^*P *< 0.05, *vs*. H+RSL (hypoxia + RS low dose).

Serum testosterone in the hypoxia group was decreased dramatically by 73.9% (*P *< 0.01) relative to control. To detect changes in the hypothalamus-pituitary-testis axis (HPT axis), an elevated serum LH was found, up to 596% (*P *< 0.01) compared to control. An increase of LH was due to the decreased inhibitory activity of serum testosterone on the HPT axis via the negative feedback mechanism. An elevated serum testosterone was responded dose-dependently to CPU86017-RS and Nif in association with a recovery of serum LH (Figure 1).

### MDA, GSH-px and LDH

**Figure 2:**
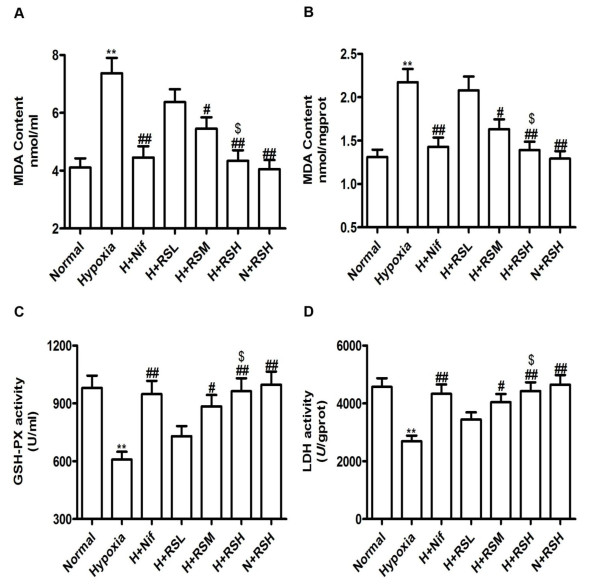
**MDA, GSH-px and LDH assay**. Production of MDA in serum and the testis was augmented and activities of GSH-px and testicular LDH were reduced by hypoxia exposure for 4 weeks in rats. These changes were attenuated by medicated (mg/kg, po) with CPU86017-RS (20, 40 and 80) and nif (10) in the last two weeks; A, Serum MDA; B. Testicular MDA; C, Serum GSH-px; D, Testicular LDH. Mean ± SD, n = 10. *P < 0.05, **P < 0.01, *vs*. Normal; ^#^*P *< 0.05, ^# #^*P *< 0.01, *vs*. Hypoxia, ^$^*P *< 0.05, *vs*. H+RSL (hypoxia + RS low dose).

After exposure to hypoxia for 4 weeks, production of MDA was increased by 79.4% and 65.8% in serum and the testis (*P *< 0.01), relative to normal, respectively. In contrast, a reduction in the activities of serum GSH-px by 37.9% and LDH in testis by 41.1% (*P *< 0.01) was found, compared to normal (Figure 2). The results indicated that the rat was suffering from oxidative insults leading to an increment in lipid peroxidation and reduced antioxidant activity both in serum and the testis, and a deficiency of energy supply in the testis was indicative of decreased activity of testicular LDH responsible for the decreased production of androgen and spermatogenesis in the seminiferous tubules. CPU86017-RS and Nif eliminated these changes significantly as compared with the hypoxia alone.

### Histological changes

**Figure 3:**
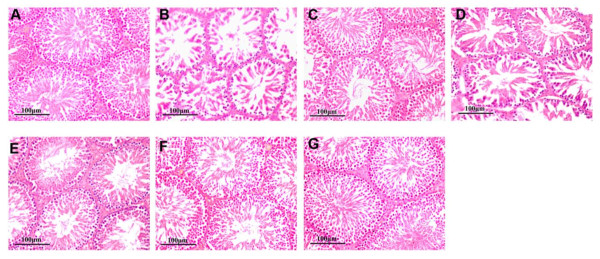
**The morphology changes in the testis**. Alterations in testis histopathology were significant following 4 weeks exposure to hypoxia and were relieved by medication with CPU86017-RS and nifedipine in the last two weeks (200×) in rats. A, Control; B, Hypoxia; C, H+Nif; D, H+RSL; E, H+RSM; F, H+RSH; G, N+RSH.

In the normal testis with H+E staining, the multilayered epithelial cells were arranged and packed orderly in the seminiferous tubules, and the reproductive epithelium tightly linked to the basement membrane of the tubules. The lumen of tubules was rich in spermatozoa and the interstitial cells were present in the gap between the tubules. Contorted structures were found in the hypoxia group by showing distorted and decreased layers of reproductive germ cells and far fewer spermatozoa leaving a large cavity at the center of lumen. The skeleton of multilayered epithelial cells and extracellular matrix was seriously disturbed in the tubules. Leydig cells disappeared at the gaps among the tubules. These changes were in agreement with low testosterone in serum. The histological abnormalities of the testis were greatly attenuated by interventions with CPU86017-RS in a dose related manner and Nif, respectively (Figure 3).

### StAR and 3-beta-HSD

**Figure 4:**
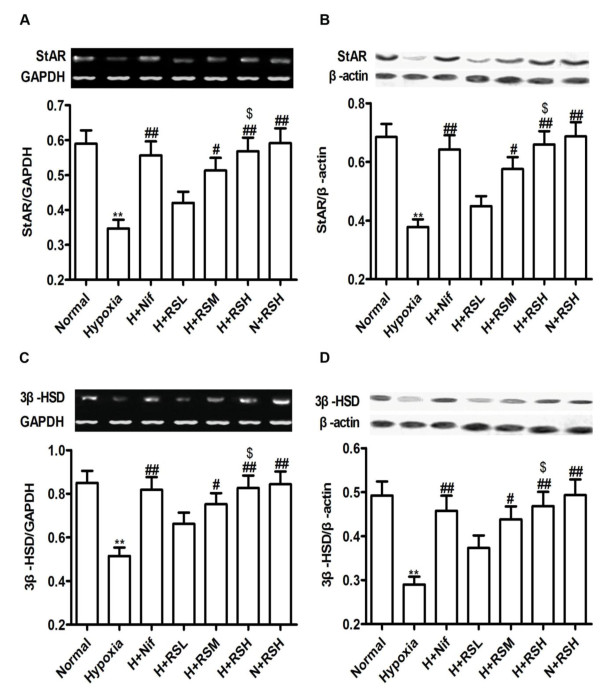
**StAR and 3-beta-HSD expression**. Downregulation of mRNA or protein expression of StAR and 3-beta-HSD was significant in rat testis suffering from hypoxia exposure for 4 weeks and was relieved by CPU86017-RS and Nif, medicated in the last 2 weeks. A, StAR mRNA; B, StAR protein; C, 3-beta-HSD mRNA; D, 3-beta-HSD protein. Mean ± SD, n = 10. *P < 0.05, **P < 0.01, *vs*. Normal; ^#^*P *< 0.05, ^# #^*P *< 0.01, *vs*. Hypoxia, ^$^*P *< 0.05, *vs*. H+RSL (RS low dose).

Responded to hypoxia, expressions of genes of StAR and 3-beta-HSD, engaging in the biosynthesis of testosterone, were downregulated in the abundance of mRNA by 40.7% (*P *< 0.01) and 38.8% (*P *< 0.01), respectively (Figure 4, A and 4C). Constantly, a reduction in protein abundance of StAR and 3-beta-HSD was evident by Western blot; down to 44.8% (*P *< 0.01) and 41.1% (*P *< 0.01), relative to normal (Figure 4, B and 4D). In response to interventions these changes were attenuated markedly (*P *< 0.01) following application of 3 doses of CPU86017-RS and Nif as compared with the hypoxia group.

### Bip, PERK and CHOP

**Figure 5:**
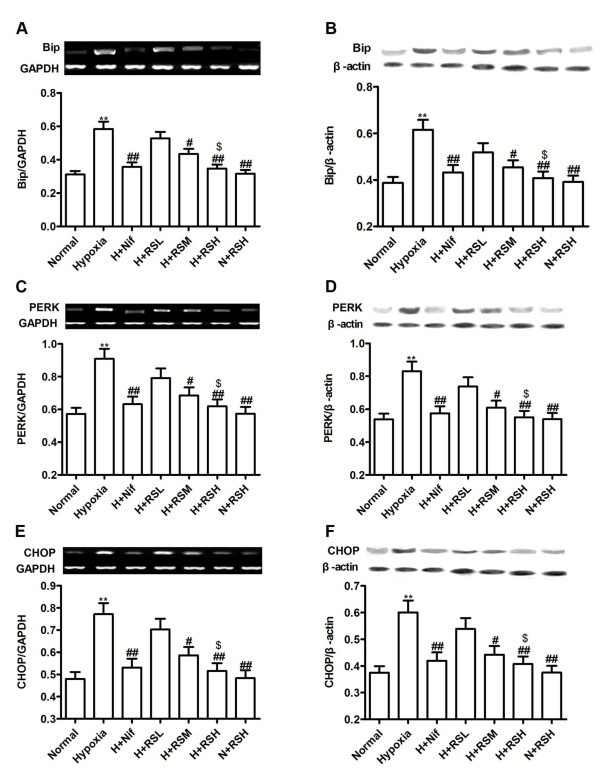
**The expressions of ER stress chaperones**. Upregulation of mRNA and protein expression of Bip, PERK and CHOP were caused by hypoxia in the testes and were attenuated by CPU86017-RS and Nif. A, Bip mRNA; B, Bip protein; C, PERK mRNA; D, PERK protein; E, CHOP mRNA; F, CHOP protein. Mean ± SD, n = 10. *P < 0.05, **P < 0.01, *vs*. Normal; ^#^*P *< 0.05, ^# #^*P *< 0.01, *vs*. Hypoxia, ^$^*P *< 0.05, *vs*. H+RSL (hypoxia + RS low dose).

It was interesting to verify if the hypoxic sufferings in the testis were characterized by an entity of inflammatory, thus, we tested the chaperones responsible for developing endoplasmic reticulum stress. An increase in mRNA expression of Bip, PERK and CHOP was signiﬁcant (P < 0.01) in the hypoxic testes, relative to normal. Protein abundances were upregulated significantly revealing the occurrence of ER stress which represented a status of chronic inflammation in the hypoxic testes. Following interventions, these abnormalities were greatly relieved, in agreement with the aforementioned findings. (Figure 5).

## Discussion

In the present study, the testis damaged by intermittent hypoxia in rats is characterized by low testosterone and high LH in serum, associated with histological changes in the testis. Under oxygen deprivation, an increase in ROS and RNS has been found as contributing factors for tissue damage [5]. Emerging data indicates that ROS serves as the requisite effector and signaling regulator in facilitating hypoxic Ca^2+ ^release [23]. Both calcium and ROS are regarded as the key messengers in the dialogue between ER and mitochondria. Ca^2+ ^overload and excess ROS genesis may synergistically contribute to dysfunction of mitochondria and ER stress and eventually cell damage and death. As we demonstrated in the present study, an application of nifedipine, a calcium antagonist, is sufficient to blunt insults in the testis caused by intermittent hypoxia. It is coincided with the findings that calcium antagonists diltiazem and nifedipine are beneficial to low temperature-induced pulmonary hypertension associated with pulmonary vascular remodeling [24], and it is also true, as demonstrated in the present study, in relieving insults in the hypoxic testis.

In response to low serum testosterone, male hypogonadism can be divided into two categories: hypogonadotrophic, with a reduced FSH and LH against low testosterone, as presented in patients with diabetes, obesity, relevant to metabolic syndrome [25]. In contrast, a low serum testosterone is frequently involved in some acute events as responded to stress, exerting less suppression on the hypothalamus-pituitary connection, as a consequence, high levels of FSH and LH occur, referred as hypergonadotrophic hypogonadism. The latter is found in the present study on exposure to hypoxia, in line with those we reported previously in isoproterenol-induced testopathy [3].

The response to intermittent oxygen deprivation manifests an entity of stress and a low level of inflammation. These changes may be linked with an increase of ROS which stimulates an increase in calcium influx to facilitate overloaded Ca^2+ ^in the cytosol. In the hypoxic pathologies in the testis, an increase in calcium influx is critical, thus, by a blockade on calcium influx, the decreased biosynthesis of testosterone in the testis and the response of the hypothalamus were blunted back to the normal levels. We suggested that Ca^2+ ^overload injury may be caused by intermittent hypoxia in the testis which was associated with chronic inflammation, and these reactions were likely found in aging, characterized as progressively increase in ROS and degenerative changes in the testes [15].

StAR is a rate-limiting factor in testosterone biosynthesis, by transporting cholesterol from outer membrane of mitochondria to its inner side [26]. In Leydig cells, StAR expression is mainly regulated by LH-mediated activation of cAMP-dependent pathways leading to transcriptional activation ultimately [27]. It is reported that testicular damage is always presented with a reduced StAR gene expression in literatures [28,29] and in our previous report [21]. The biosynthesis of androgen is completed in serial reactions, in which 3-beta-HSD catalyzes the conversion of dehydroepiandrosterone to androstenedione in mitochondria, thereafter, the process of biosynthesis of testosterone is continued while moving into the ER in Leydig cells. The two enzymes presented a state of downregulation strongly indicating poor testosterone production in Leydig cells, eventually leading to a great decline in serum testosterone following hypoxia exposure [30].

ER stress has been expanded dramatically in explaining the entity of disease as low levels of inflammatory status in many diseases, and oxidative stress, calcium signaling and inflammatory factors have been recognized as the key messengers for inducing ER stress [16]. Regarding triggered factors in tissues, elevated pro-inflammatory cytokines, an excess of endothelin-1, hyperglycemia, hypercholesterolemia and disturbance of Ca^2+ ^homeostasis are active in initiating ER stress. The UPR defined as a defensive response in its initial stage of ER stress, leading to a reduction in the ER workload by translational attenuation, then, the maladaptive response in the ER can be overcome. However, while risk factors, such as an excess of ROS genesis are persistent due to prolonged oxygen deprivation, more calcium influx into the cytoplasm can be seen in hypoxic pulmonary hypertension [11]. This situation could be reproduced in the hypoxic testis. In fact, in the present study, upregulation of Bip, identical to the 78 kDa glucose regulated protein (GRP78), serves as a central regulator of ER stress and indicates its dissociation from the binding sites of the following chaperones ATF6, PERK and IRE1. Some studies using solid tumors showed that hypoxia induces Bip expression via activated ER stress to improve protein aggravation [31]. As demonstrated in the present study, a normal expression rather than an upregulated expression of Bip is essential in keeping the testis free from hypoxic insults.

PERK, a type I transmembrane kinase, can be activated by auto-phosphorylation and auto- dimerization following dissociation with Bip [32]. Activated PERK may rescue the cells from ER overload by phosphorylating eukaryotic initiation factor 2α (eIF2α) at Ser 51, which inhibits mRNA translation in the nucleus. Cell protection in the hypoxic testes could be achieved by upregulation of PERK through reducing translation contributing to reduction of critical regulatory proteins which promotes activation of transcription factors such as NFκ-B under cellular stress [33]. Important consequence of PERK- eIF2α pathway could take place in the testis damaged by hypoxia exposure, the same as those showed in diabetic nephropathy which is characterized by upregulation of PERK in renal tissue [34].

ER stress, a double-edged sword, is beneficial at first, and appears to be harmful while upregulation of CHOP/GADD153 occurs. CHOP conducts the transition from the protective phase to the death-promoting phase of the UPR under intermittent hypoxia. Death executor CHOP induces apoptosis by promoting protein synthesis and oxidation in the stressed ER [35]. Many pathologies in the hypoxic testis are aggravated via CHOP-induced apoptosis. Our results indicate that significantly upregulated CHOP in the testis on chronic hypoxic exposure represents a severe damage to the testicular tissue due to ER stress. An increase in CHOP chaperone facilitates the appearance of apoptosis and testicular cell dysfunction possibly by suppressing Bcl-2 through JNK/CHOP/DR5 signaling [36], and stimulating Bim (BH3-only protein) via protein phosphatase 2A-mediated dephosphorylation and CHOP-C/EBPα-mediated transcriptional induction [37] and an enhancement of PUMA promoter through ATF4-CHOP pathway [38]. CHOP associated with severe testis damage was suppressed by CPU87017-RS and Nif.

The alteration of Bip, PERK and CHOP confirms that ER stress mediates the pathogenesis of testopathy under hypoxic exposure. Interestingly, inhibition of the Ca^2+ ^influx by nifedipine is significant in this regards, indicating the importance of blockade on Ca^2+ ^channels in attenuating ER stress in the hypoxic testis. Therefore, an alleviation of ER stress by CPU86017-RS is probably relevant to its calcium influx blocking activity in the hypoxic testis.

### Limitations

As we revealed in the present study, the restricting of calcium influx is crucial in attenuating the testicular lesions by intermittent hypoxia. CPU86017-RS suppresses the calcium channels significantly [11,18]. We believe that CPU86017-RS and nifedipine exert beneficial effects relevant to the calcium antagonist activity in the testis, thereafter; a reduction in oxidative stress is the consequence by suppressing NADPH oxidase in the testis. However, direct suppression on NADPH oxidase and ROS genesis in Leydig cells by calcium influx restricting effects of CPU86017-RS and Nif are not offered in the present study. Downregulation of androgen biosynthesizing genes associated with exacerbated chaperones of ER stress in the Leydig cells is presumably modulated by calcium influx, which is needed for further exploration in the testicular dysfunction suffering from hypoxia.

## Conclusion

Upregulation of Bip, PERK and CHOP is the major event in hypoxia testes in rats, contributing to low expression of StAR and 3-beta-HSD responsible for compromised testosterone biosynthesis. These changes are likely the consequence to an activation of an increased calcium influx in the testicular cells under oxygen deprivation. CPU86017-RS is potential for use in relieving hypoxia-induced testicular dysfunction through alleviating ER stress via its calcium antagonism. A restriction of calcium influx which modulates ER stress and the StAR and 3-beta-HSD gene expression is worth to explore further in Leydig cells.

## Abbreviations

3-beta-HSD: 3-beta-hydroxysteroid dehydrogenase; Bip: Immunoglobulin heavy chain binding protein; CHOP: C/EBP homologous protein; CPU86017-RS: (+)-7R, 13aS-p-chlorobenzyltetrahydroberberine chloride; ER: Endoplasmic reticulum; ER stress: Endoplasmic reticulum stress; FSH: follicle-stimulating hormone; HPH: hypoxic pulmonary hypertension; HPT axis: hypothalamus-pituitary-testis axis; LH: luteinizing hormone; PERK: Double-strand RNA-activated protein kinase-like ER kinase; RNS reactive nitrogen species; ROS: Reactive oxygen species; StAR: Steroidogenic acute regulatory protein; UPR: Unfolded protein response.

## Competing interests

The authors declare that they have no competing interests.

## Authors' contributions

DZD and YD conceived of the study, coordinated all studies and helped to draft and revise the manuscript. GLL and FY conducted biochemistry assay and the quantification of gene expression and drafted the manuscript. GLZ was responsible for conducting the hypoxic exposure of rats. CZ synthesized CPU86017-RS. All authors read and approved the manuscript.
